# Endothelial Differentiation Gene-1, a New Downstream Gene Is Involved in RTEF-1 Induced Angiogenesis in Endothelial Cells

**DOI:** 10.1371/journal.pone.0088143

**Published:** 2014-02-10

**Authors:** Ping He, Melissa J. Philbrick, Xiaojin An, Jiaping Wu, Angela F. Messmer-Blust, Jian Li

**Affiliations:** 1 Department of Gerontology of Union Hospital, Tongji Medical College of Huazhong University of Science and Technology, Wuhan, China; 2 Cardiovascular Institute, Beth Israel Deaconess Medical Center, Harvard Medical School, Boston, Massachusetts, United States of America; William Harvey Research Institute, Barts and The London School of Medicine and Dentistry, Queen Mary University of London, United Kingdom

## Abstract

Related Transcriptional Enhancer Factor-1 (RTEF-1) has been suggested to induce angiogenesis through regulating target genes. Whether RTEF-1 has a direct role in angiogenesis and what specific genes are involved in RTEF-1 driven angiogenisis have not been elucidated. We found that over-expressing RTEF-1 in Human dermal microvascular endothelial cells-1 (HMEC-1) significantly increased endothelial cell aggregation, growth and migration while the processes were inhibited by siRNA of RTEF-1. In addition, we observed that Endothelial differentiation gene-1 (Edg-1) expression was up-regulated by RTEF-1 at the transcriptional level. RTEF-1 could bind to Edg-1 promoter and subsequently induce its activity. Edg-1 siRNA significantly blocked RTEF-1-driven increases in endothelial cell aggregation in a Matrigel assay and retarded RTEF-1-induced endothelial cell growth and migration. Pertussis Toxin (PTX), a Gi/Go protein sensitive inhibitor, was found to inhibit RTEF-1 driven endothelial cell aggregation and migration. Our data demonstrates that Edg-1 is a potential target gene of RTEF-1 and is involved in RTEF-1-induced angiogenesis in endothelial cells. Gi/Go protein coupled receptor pathway plays a role in RTEF-1 driven angiogenesis in endothelial cells.

## Introduction

Related Transcriptional Enhancer Factor 1 (RTEF-1), also known as TEAD4 (TEA domain family member 4), is a member of the Transcription Enhancer Factor family, and plays important roles in a variety of physiological and pathological conditions. RTEF-1 targets the promoters of many genes and shares a highly conserved domain capable of binding to the MCAT element CATN(T/C)(T/C) [1], [2] in the promoter region of genes expressed in endothelial [3], cardiac [4], skeletal and smooth muscle cells [5], as well as myofibroblasts [6]. In endothelial cells, RTEF-1 is involved in the stimulation of angiogenesis under hypoxia via transcriptional regulation of its target genes [7]. RTEF-1 is shown to transcriptionally regulate Hypoxia inducible factor (HIF)-1 and accelerate recovery rates from hind limb ischemic injury [3]. In addition, RTEF-1 impacts the Fibroblast growth factor (FGF)/FGFR system through the eNOS pathway in the regulation of angiogenesis and vasodilation [8]. We have recently found that endothelial specific RTEF-1-deficient mice lack the ability to form normal capillary networks suggesting that a loss of RTEF-1 signaling leads to the disintegration of the mature vasculature and that RTEF-1 is required for endothelial connections and capillary network formation [9]. However, the direct effect of RTEF-1 on angiogenesis in endothelial cells and new target genes that might be involved in RTEF-1 driven angiogenesis has not been fully understood.

Endothelial differentiation gene 1 (Edg-1), also known as sphingosine-1-phosphate receptor 1 (S1PR1 or S1P1) is a G-protein-coupled receptor. It binds the ligand sphingosine-1-phosphate (S1P) with high affinity and high specificity and is suggested to be involved in the processes that regulate the differentiation of endothelial cells. Activation of this receptor by S1P induces cell–cell adhesion [10], migration and proliferation in endothelial cells [11]. Edg-1^−/−^ mice exhibit embryonic hemorrhage leading to intrauterine death between E12.5 and E14.5 due to a deficiency in vascular maturation [12]. These findings suggest that Edg-1 plays an important role in angiogenesis. However, transcription factors that regulate Edg-1 in angiogenesis have not been elucidated.

Here, we report that the transcription factor RTEF-1 is involved in enhancing angiogenesis in endothelial cells. Furthermore, we show that RTEF-1 regulates Edg-1 gene expression as a transcriptional activator upon binding to Edg-1 promoter. In addition, the angiogenesis enhanced by RTEF-1 is associated with the Edg-1 and Pertussis Toxin (PTX)-sensitive Gi/Go protein pathway.

## Materials and Methods

### Ethics Statement

The animal study was carried out in strict accordance with the recommendations in the Guide for the Care and Use of Laboratory Animals of the National Institutes of Health. The protocol was approved by the Institutional Animal Care and Use Committee at Beth Israel Deaconess Medical Center (049-2011).

### Cell Culture

Human dermal microvascular endothelial cells-1 (HMEC-1; Center of Disease Control) were cultured in MCDB-131 (Invitrogen, Carlsbad, CA) containing 10% fetal bovine serum, 10 ng/ml epidermal growth factor, 1 µg/ml hydrocortisone and 2 mM L-glutamine. HEK293 (human embryonic kidney 293; ATCC) and HEK293T (a derivative of HEK293 which constitutively expresses the simian virus 40 large T antigen; ATCC) cells were cultured in Dulbecco’s modified Eagle’s medium (Invitrogen, Carlsbad, CA) with 10% fetal bovine serum.

### Generation of RTEF-1 Transgenic Mice

RTEF-1 transgenic mice were generated at the BIDMC Transgenic Core Facility using the vascular endothelial (VE)-cadherin promoter to drive endothelial-specific expression of human RTEF-1 [13]. RTEF-1 transgenic mice were genotyped by PCR as described previously [14]. A conditional knockout (KO) line of RTEF-1 was generated by crossing homozygous *TEAD4*lox/lox mice (a gift from Dr. Andres Buonanno, NICHHD, NIH) with transgenic mice expressing *Cre* recombinase under control of the endothelial cell-specific Tie2 promoter/enhancer (a gift from Dr. Anthony Rosenzweig, BIDMC) [15]. Mice were screened by PCR to verify germline transmission, using primers P1, P2, P3 and/or P4 as described previously [16].

### Retroviral Transduction

The stable over-expression of RTEF-1 in HMEC-1 cells was performed as previously described [3]. In brief, the coding sequence of RTEF-1 (NM_003213) was subcloned into the pBMN-GFP vector (Orbigen, San Diego, CA). In 10-cm dishes, 6×10^6^ HEK293T cells were transfected with pBMN-GFP-RTEF-1, pMD-VSVG, pJK3, or pCMV-tat using Polyethyleneimine (Polysciences, Warrington, PA). After 48 hours transfection, virus-containing medium was collected, filtered through a 0.45-µm filter, and used as a transducer for HMEC-1 cells. After transfection, cells were selected with puromycin (250 ng/ml).

### Small Interfering RNA Transfection

Small interfering RNA (siRNA) encoding human RTEF-1 or Edg-1 (Genpharma Shanghai, China) at a final concentration of 50 nM was transfected using Lipofectamine 2000 (Invitrogen, Carlsbad, CA) and confirmed by real- time PCR and western blotting. Two duplexes of RTEF-1 siRNA (5′-GGG CAG ACC UCA ACA CCA ATT-3′, 5′-UUG GUG UUG AGG UCU GCC CAG-3′ and 5′- ACC CAA GAU GCU GUG UAU UTT- 3′, 5′- AAU ACA CAG CAU CUU GGG UTT-3′) and two duplexes of Edg-1 siRNA (5′- GCU AUA UCA CAA UGC UGA ATT, 5′- UUC AGC AUU GUG AUA UAG CTT-3′ and 5′- GCC CAC UUU AUC UAA AUG ATT-3′, 5′- UCA UUU AGA UAA AGU GGG CTT-3′) were used in the experiment. A duplex of RNA(5′- UUC UCC GAA CGU GUC ACG UTT-3′, 5′- ACG UGA CAC GUU CGG AGA ATT-3′) that is not targeted to any human gene was used as a negative control. Briefly, a master mix of Lipofectamine 2000 was diluted with 1 ml of OPTI-MEM (Invitrogen, Carlsbad, CA) and incubated for 5 min. The Lipofectamine 2000 dilution was added to the siRNA dilution, incubated for 20 min, and added drop-wise to the cells. Five hours after transfection, medium was changed and the cells were allowed to recover overnight. Forty-eight hours after transfection, cells were harvested for mRNA and protein dectection or followed by Matrigel, migration and cell growth assays as designed.

### RNA and Protein Analyses

Total RNA was extracted from cells or lungs of mice that were euthanized by carbon dioxide. A total of 2.0 µg of RNA was reverse-transcribed using the High Capacity cDNA Reverse Transcription kit (Applied Biosystems, Carlsbad, CA) with random primers according to the manufacturer’s protocol. Quantitative real-time PCR amplification was done using SYBR Green Master Mix (Applied Biosystems, Carlsbad, CA) according to the manufacturer’s instruction. Gene expression was analyzed with the primers for Edg-1 (5′- CTCCACAACGGGAGCAATAAC-3′ and 5′- CAGCGCACTGATGCAGTTC-3′), RTEF-1 (5′-CCACGAAGGTCTGCTCTTTC-3′ and 5′-CTCACTGGCTGACACCTCAA-3′) and Actin (5′- TCTTTAATGTCACGCACGATT-3′ and 5′- TCACCCACACTGTGCCCAT-3′).

Proteins were isolated from cells or tissue samples lysed in cold RIPA buffer (Boston BioProducts, Worcester, MA) supplemented with a cocktail of protease inhibitors (Roche Applied Science, Branford, CT). Protein concentration was determined with the DC Protein Standard Assay (Bio-Rad, Hercules, CA). Samples were subjected to 10–12% SDS-PAGE, transferred to polyvinylidene fluoride membranes (Millipore, Billerica, MA) and subsequently blocked in Tris-buffered saline-Tween 20 containing 8% nonfat milk. The membranes were then incubated with the indicated primary antibodies: anti-Edg-1 (NOVUS Biologicals, Littleton, CO, 1∶1000), anti-RTEF-1 (Genemed Synthesis Inc, San Antonio, TX; 1∶10,000) and anti-Vinculin (Sigma-Aldrich, St. Louis, MO; 1∶100,000), respectively, followed by incubation with horseradish peroxidase-conjugated secondary antibodies anti-rabbit IgG with 1∶3000 dilution (Calbiochem, La Jolla, CA) or anti-mouse IgG with 1∶2000 dilution (Vector Labs, Burlingame, CA). Blots were developed using the chemiluminescence detection system according to the instructions of the manufacturer (Thermo Fisher, Pittsburgh, PA).

### Matrigel Analysis

Growth Factor-Reduced BD Matrigel Matrix (BD Biosciences, San Jose, CA) was coated on a pre-chilled 24-well culture plate on ice. After Matrigel solidification for 30 minutes, RTEF-1 over-expression (RTEF-1 o/e) HMEC-1 and pBMN-GFP over-expression (control) HMEC-1 cells were plated at 10^5^ cells per well. The extent of network formation was observed and photographed at 6 h and 24 h of culture in Matrigel. In a second set of experiments, RTEF-1 siRNA or corresponding negative control siRNA (50 nmol/ml each) transfected control HMEC-1 cells and Edg-1 siRNA or corresponding negative control siRNA (50 nmol/ml each) transfected RTEF-1 o/e HMEC-1 cells and control HMEC-1 cells were plated in Matrigel and the capillary network was observed at 24 h. In a tertiary experiment, the capillary network formation was tracked at 24 h Matrigel in RTEF-1 o/e HMEC-1 or control HMEC-1 with or without PTX (200 ng/mL, Tocris Biosciences, Minneapolis, MN) treatment. Tubule length ratio, branch number per field and aggregation area were analyzed using Image J software.

### Migration Analysis

Cell migration in RTEF-1 siRNA (50 nmol/ml) treated control HMEC-1 cells and Edg-1 siRNA (50 nmol/ml) treated RTEF-1 o/e HMEC-1 cells and control HMEC-1 cells were compared to their corresponding negative siRNA transfected controls. The comparison was also performed in RTEF-1 o/e HMEC-1 cells and control HMEC-1 cells treated with or without PTX (200 ng/mL). The cell migration rate was measured using the wound healing assay. Briefly, equal numbers (1×10^5^) of RTEF-1 o/e HMEC-1 and control HMEC-1 cells were plated in 12-well plates and serum-starved in 0.5% serum medium for 24 hours. The monolayer of cells was wounded by manual scratching with a 200 µl pipette tip and the cells were then cultured in complete growth medium. Matched wound regions were photographed with a Nikon microscope using phase contrast at 0 h and 12 h migration. The migration rate was assessed by the quotient of the cell migration distance divided by the original scratched width.

### Cell Growth Assay

Crystal violet assay was used to detect cell growth. A total of 10^4^ cells of control HMEC-1 and RTEF-1 o/e HMEC-1 treated with or without RTEF-1 siRNA (50 nmol/ml) and Edg-1 siRNA (50 nmol/ml) were plated in each well of a 24-well plate. At 5 h and on 2, 3, and 4 days after plating, cells were fixed in 100% ethanol and subsequently stained with 0.1% crystal violet dissolved in 10% ethanol. After staining and thorough washing, the dye was extracted with 10% acetic acid, and absorbance was measured at 590 nm.

### Immunofluorescence

Lungs were removed from transgenic and littermate control mice euthanized by carbon dioxide, embedded in O.C.T. compound (Sakura Finetek USA Inc., Torrance, CA), and frozen at −80°C. Tissues were sectioned with a Cryostat CM Model 3050S-3-1-1 (Leica, Wetzlar, Germany), fixed in 4% paraformaldehyde, and stained with antibodies against Edg-1 (Novus Biologicals, Littleton, CO) with a 1∶1000 dilution and Lectin (Sigma-Aldrich, St. Louis, MO) with a 1∶500 dilution followed by incubation with goat anti-rabbit TRITC and FITC antibody (Santa Cruz, Santa Cruz, CA) at a dilution of 1∶500. Immunofluorescence-stained sections were visualized with a DM5000B upright microscope (Leica, Wetzlar, Germany).

### Promoter Activity and Chromatin Immunoprecipitation (ChIP)

A construct containing nucleotide fragment (−831 bp to 174 bp of human sequence at GeneBank: NM_001400.4) encompassing basal elements of the human Edg-1 promoter was obtained from Switchgear Genomics (Menolo Park, CA). HEK293 cells were transfected with the construct and different doses of pXJ40/RTEF-1 and pXJ40 (gifted from Dr. Alexandre Stewart, University of Ottawa) using Polyethyleneimine. The addition of pXJ40 was used to maintain the same cDNA background. Luciferase activity was determined using the dual luciferase assay system (Switchgear Genomics, Menolo Park, CA) 24 hours after transfection. Chromatin immunoprecipitation was performed with the ChIP-IT Express Kit (Active Motif, Carlsbad, CA) according to the manufacturer’s instructions. The Edg-1 and Actin promoters were amplified with the primer pairs 5′-AAA CCC CTC GCC CAA CAC CAA-3′, 5′-GCG AGA GGT ACG GAG GAG ACA AGC-3′, and 5′- TCT TTA ATG TCA CGC ACG ATT-3′, 5′- TCA CCC ACA CTG TGC CCA T-3′, respectively. Edg-1 primers were designed to the putative MCAT element binding site for RTEF-1 and the amplification sequence was −595 bp to −418 bp from the transcription start site.

### Statistical Analysis

Results are expressed as the mean value±SD. Student’s *t* test was used for two samples test and multiple comparisons among three or more groups were carried out by one-way ANOVA. A value of *P*<0.05 was considered significant.

## Results

### RTEF-1 Promotes Endothelial Cell Aggregation, Migration and Cell Growth

**Figure 1:**
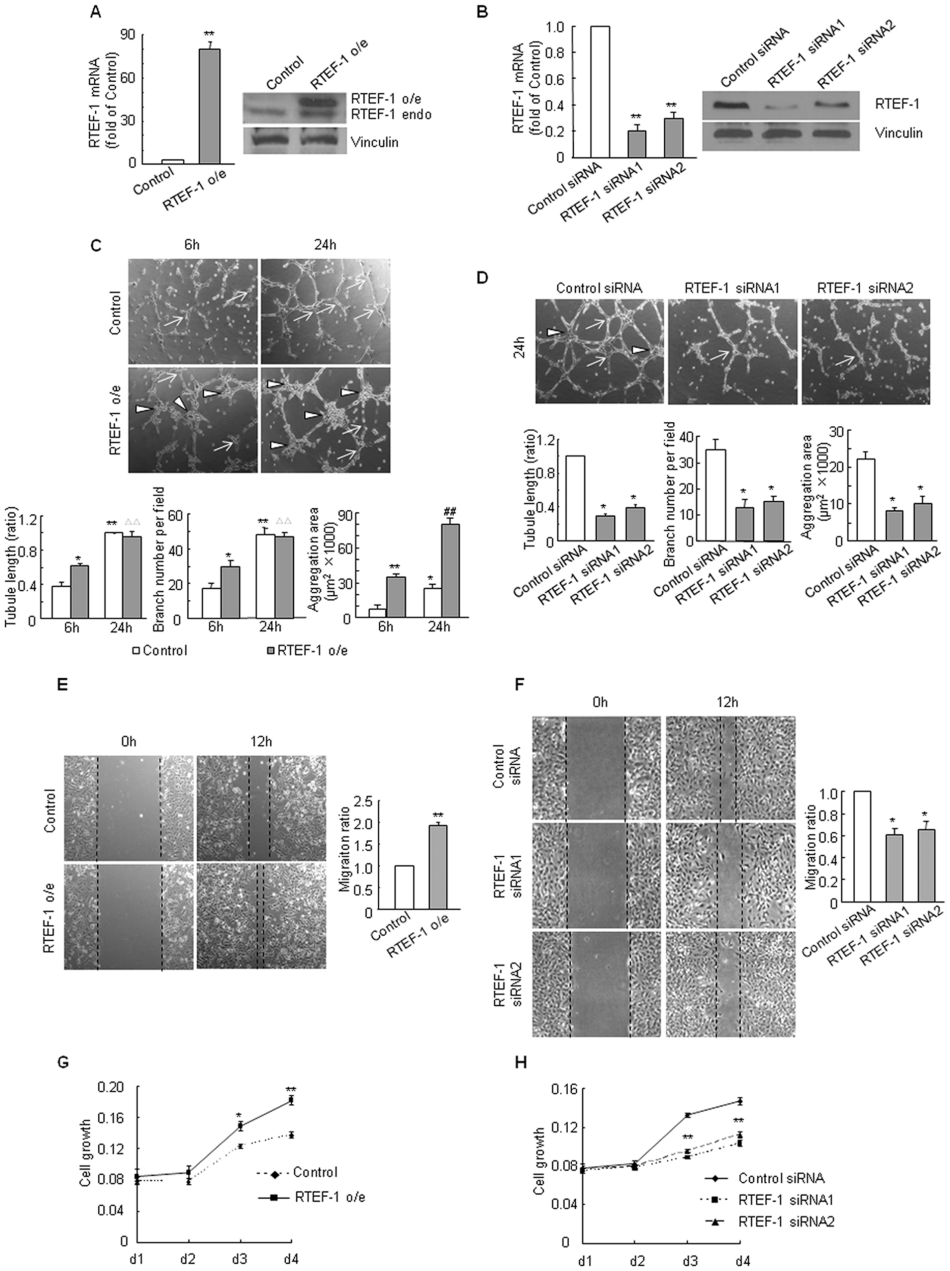
RTEF-1 induces angiogenesis in endothelial cells. **A.** RTEF-1 was over-expressed in HMEC-1. RTEF-1 mRNA (left) and protein (right) were detected in control HMEC-1 and RTEF-1 o/e HMEC-1. (** = *p*<0.01, *vs* control HMEC-1) **B.** RTEF-1 expression was knocked down by RTEF-1 siRNA in control HMEC-1. (** = *p*<0.01, *vs* control HMEC-1; left: mRNA; right: protein) **C.** RTEF-1 enhanced endothelial cell connections. The tubule length (left) and branch number (middle) were increased by RTEF-1 at 6 h in Matrigel compared to control. Notably, tube formation was more apparent at 24 h than at 6 h in Matrigel. The aggregation area (right) were increased by RTEF-1 both at 6 h and 24 h in Matrigel and pronounced at 24 h in Matrigel. (* = *p*<0.05, *vs* control 6 h HMEC-1; ** = *p*<0.01, *vs* control 6 h HMEC-1; ΔΔ = *p*<0.01, *vs* RTEF-1 o/e 6 h Matrigel; ##* = p*<0.01, *vs* control 24 h HMEC-1; Triangle: aggregation area; Arrow: branch) **D.** RTEF-1 siRNA decreased endothelial cell connections. The tubule length (left), branch number (middle) and aggregation area (right) in Matrigel assay were significantly lowered by RTEF-1 siRNA. (* = *p*<0.05, *vs* negative control siRNA; Triangle: aggregation area; Arrow: branch) **E.** RTEF-1 increased endothelial cell migration. (** = *p*<0.01, *vs* control HMEC-1) **F.** RTEF-1 siRNA retarded endothelial cell migration. (* = *p*<0.05, *vs* control HMEC-1) **G.** RTEF-1 increased endothelial cell growth. (* = *p*<0.05, *vs* control HMEC-1; ** = *p*<0.01, *vs* control HMEC-1) **H.** RTEF-1 siRNA inhibited endothelial cell growth (** = *p*<0.01, *vs* control HMEC-1). The results were quantified based on three independent experiments and are presented as mean±S.D.

To determine the direct effect of RTEF-1 on angiogenesis in endothelial cells, we constructed the stable over-expressing RTEF-1 (RTEF-1 o/e) HMEC-1 (Figure 1A) and the transient RTEF-1 siRNA-transfected HMEC-1 (Figure 1B). In the Matrigel assay, RTEF-1 o/e HMEC-1 cells showed a significant cell aggregation both at 6 h and 24 h and tube formation mostly at 6 h (Figure 1C) while RTEF-1 siRNA-transfected HMEC-1s in which endogenous RTEF-1 was knocked down attenuated the formation of normal capillary network at 24 h (Figure 1D). In addition, RTEF-1 o/e HMEC-1 demonstrated a rapid migration in the scratch assay (Figure 1E) and a rising cell growth in the crystal violet assay (Figure 1G) whereas RTEF-1 siRNA retarded endothelial cell migration (Figure 1F) and growth (Figure 1H). The observation indicated that RTEF-1 promotes angiogenesis in endothelial cells.

### RTEF-1 Regulates Edg-1 Gene Expression *in vitro* and *in vivo*

**Figure 2:**
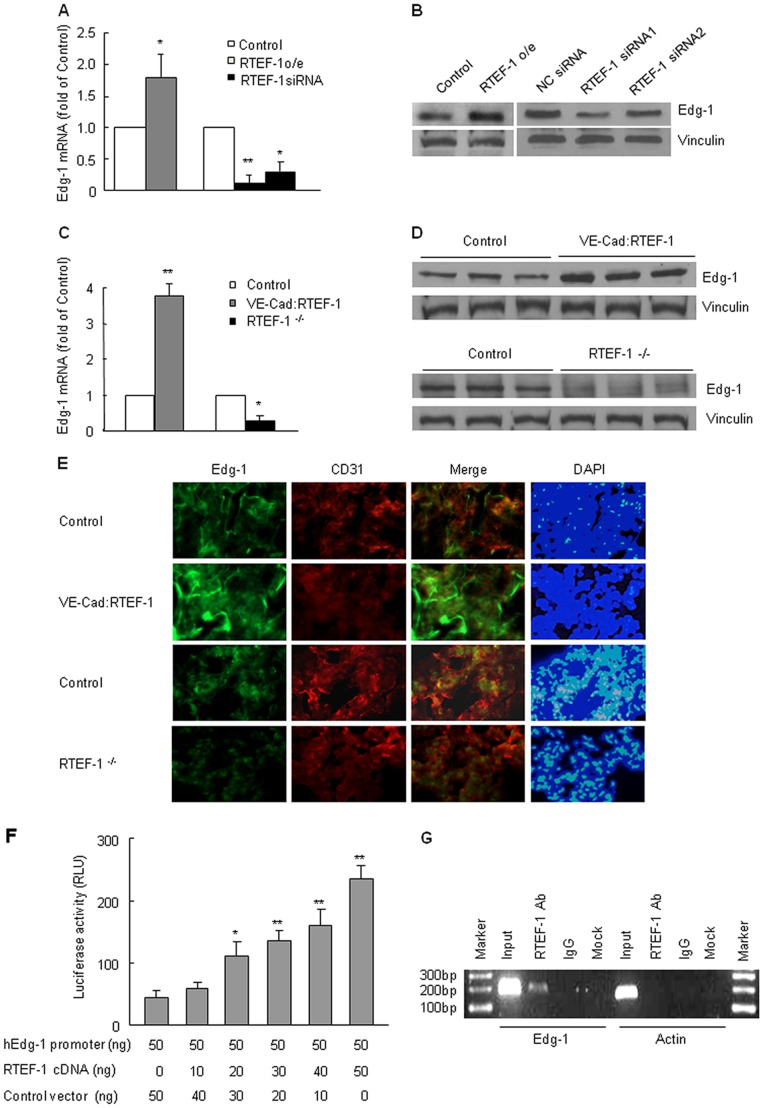
Edg-1 is a potential target gene of RTEF-1 *in vitro* and *in vivo*. **A.** Edg-1 mRNA levels were significantly increased in RTEF-1 o/e HMEC-1 and decreased in RTEF-1 knockdown control HMEC-1. (* = *p*<0.05; ** = *p*<0.01, *vs* control HMEC-1) **B.** Edg-1 protein levels showed similar results to mRNA levels when RTEF-1 was over-expressed (left panel) or knocked down (right panel). **C.** Edg-1 mRNA expression level in the lung was increased in VE-Cad: RTEF-1 mice and decreased in RTEF-1^−/−^ mice (* = *p*<0.05; ** = *p*<0.01, *vs* control mice). **D.** Immunoblot analysis of lungs from both VE- Cad: RTEF-1 mice and RTEF-1^−/−^ mice. VE-Cad: RTEF-1 mice showed an increase in Edg-1 expression, while RTEF-1^−/−^ mice showed a decrease in Edg-1 expression when compared to their littermate controls. **E.** Lung tissues from VE-Cad: RTEF-1 mice, RTEF-1^−/−^ mice and their littermate controls were stained with anti-Edg-1 and anti-CD31 antibodies. **F.** Edg-1 full length promoter was transiently co-transfected with different concentrations of RTEF-1, and luciferase activity was examined. Edg-1 promoter activity was shown to be up-regulated in an RTEF-1 dose-dependent manner. (* = *p*<0.05; ** = *p*<0.01). **G.** ChIP assays were performed by immunoprecipitating chromatin from HMEC-1 cells with control IgG or anti-RTEF-1 antibody and followed by RT-PCR. The results were quantified from three independent experiments and are presented as mean±S.D.

In a DNA microarray data from human umbilical vein endothelial cells (HUVEC), we found that Edg-1 was significantly down-regulated following RTEF-1 knockdown by RTEF-1 siRNA (*p* = 0.0002, *vs* negative control siRNA treated HUVEC) (Figure S1). To test our hypothesis that Edg-1 might be a potential target gene of RTEF-1, RTEF-1o/e HMEC-1 as well as RTEF-1 knockdown HMEC-1 by RTEF-1 siRNA was examined for Edg-1 expression. Edg-1 mRNA increased to 1.80 fold in RTEF-1o/e HMEC-1 cells and decreased to 0.13 fold and 0.30 fold in RTEF-1 knockdown HMEC-1 cells compared to their corresponding controls (Figure 2A). Edg-1 protein expression in HMEC-1 cells with or without RTEF-1 showed a similar trend to that of Edg-1 mRNA (Figure 2B). Moreover, expression of Edg-1 in lung tissue dramatically increased in VE-Cad: RTEF-1 mice and decreased in RTEF-1^−/−^ mice compared to their littermate controls (mRNA expression shown in Figures 2C and protein expression shown in 2D). We further confirmed that Edg-1 was localized in the endothelium by co-staining with antibodies against CD31 and Edg-1 in the lungs of control and transgenic mice (Figures 2E) and the expression of Edg-1 was apparently associated with the RTEF-1 gene level in these mice.

To determine whether Edg-1 is regulated by RTEF-1 on a transcriptional level, the Edg-1 promoter was co-transfected with RTEF-1 cDNA and subjected to luciferase assay. The Edg-1 promoter exhibited an increased activity in an RTEF-1 dose-dependent manner, with a maximum 4.0 fold enhancement (Figure 2F). The physical interaction of RTEF-1 with the Edg-1 promoter was examined by ChIP assay in RTEF-1 o/e HMEC-1. As shown in Figure 2G, a specific DNA fragment was detected in RTEF-1 immunoprecipitated chromatin. Together, these data indicated that RTEF-1 up-regulated Edg-1 expression in endothelial cells by binding with Edg-1 promoter and increasing Edg-1 promoter activity.

### Edg-1 Takes Part in RTEF-1-driven Endothelial Cell Aggregation, Migration and Growth

**Figure 3:**
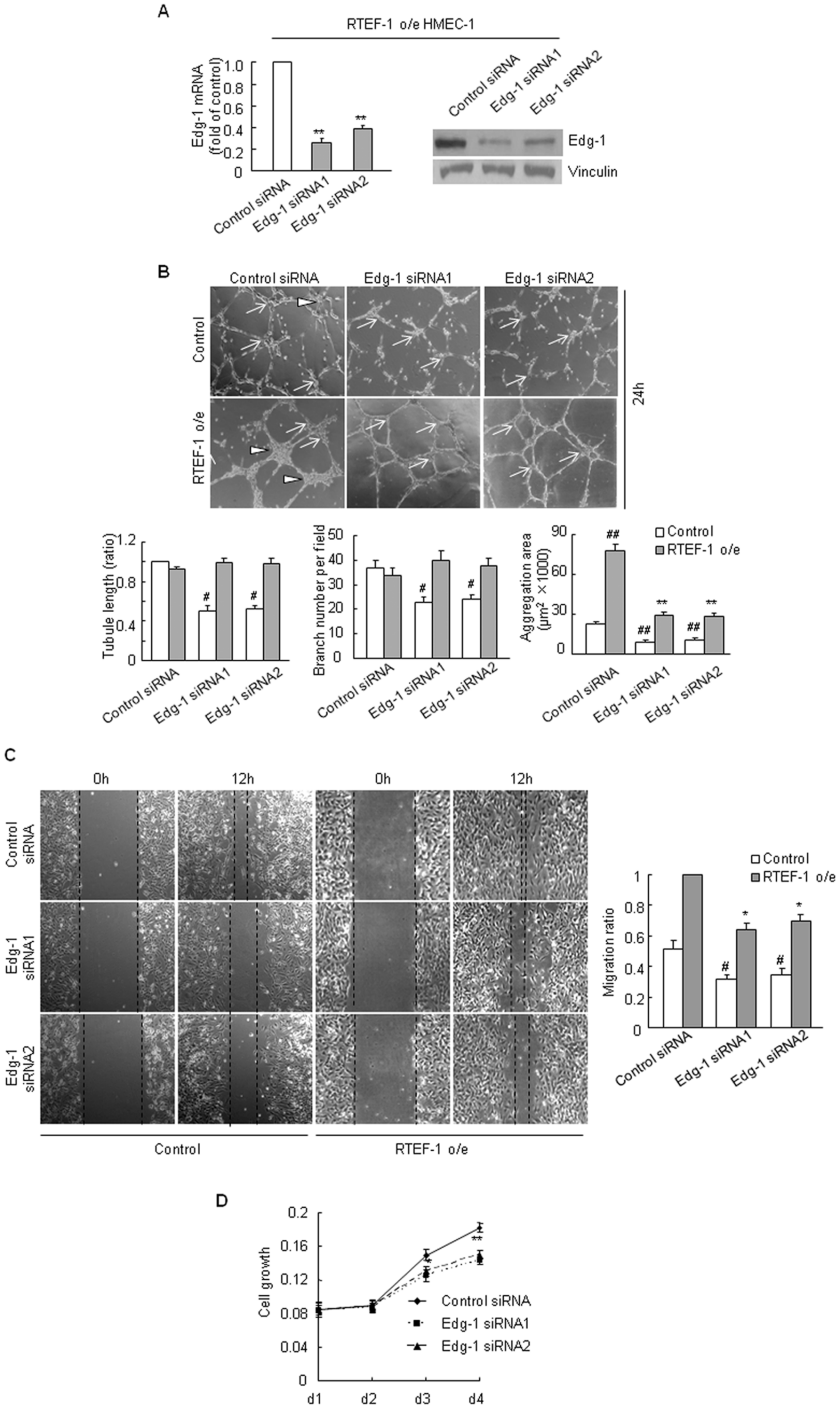
Edg-1 is involved in RTEF-1 driven angiogenesis in endothelial cells. **A.** Edg-1 expression was knocked down by Edg-1 siRNA. (** = *p*<0.01, *vs* control siRNA treated RTEF-1 o/e HMEC-1) **B.** Edg-1 siRNA inhibited RTEF-1 driven endothelial cell aggregation. (# = *p*<0.05, *vs* negative control siRNA treated control HMEC-1; ** = *p*<0.01, *vs* negative control siRNA treated RTEF-1 o/e HMEC-1; ## = *p*<0.01, *vs* negative control siRNA treated control HMEC-1; Triangle: aggregation area; Arrow: branch) **C.** Edg-1 siRNA inhibited RTEF-1 driven endothelial cell migration. (# = *p*<0.05, *vs* negative control siRNA treated control HMEC-1; * = *p*<0.05, *vs* negative control siRNA treated RTEF-1 o/e HMEC-1) **D.** Treatment with Edg-1 siRNA attenuated cell growth in RTEF-1 o/e HMEC-1 (* = *p*<0.05; ** = *p*<0.01, *vs* negative control siRNA). The results were quantified based on three experiments and are presented as mean±S.D.

To determine whether Edg-1 is involved in RTEF-1 induced angiogenesis in endothelial cells, we transiently transfected Edg-1 siRNA into RTEF-1 o/e HMEC-1 and control HMEC-1 and observed endothelial cell aggregation, migration and growth. As shown in Figure 3B, Edg-1 siRNA significantly decreased the aggregation area both in RTEF-1 o/e HMEC-1 and control HMEC-1, while the inhibitory effect on tube formation was obvious in control HMEC-1 but not in RTEF-1 o/e HMEC-1. Knockdown of Edg-1 also retarded cell migration in both RTEF-1 o/e HMEC-1 and control HMEC-1 compared with negative control siRNA (Figure 3C). Crystal violet assay demonstrated that RTEF-1 o/e HMEC-1 cell growth had been inhibited by Edg-1 siRNA after culture for 2 days (Figure 3D). These results showed that Edg-1 participates in RTEF-1 stimulated angiogenesis in endothelial cells.

### PTX Inhibited RTEF-1 Driven Endothelial Cell Tube Formation and Migration

**Figure 4:**
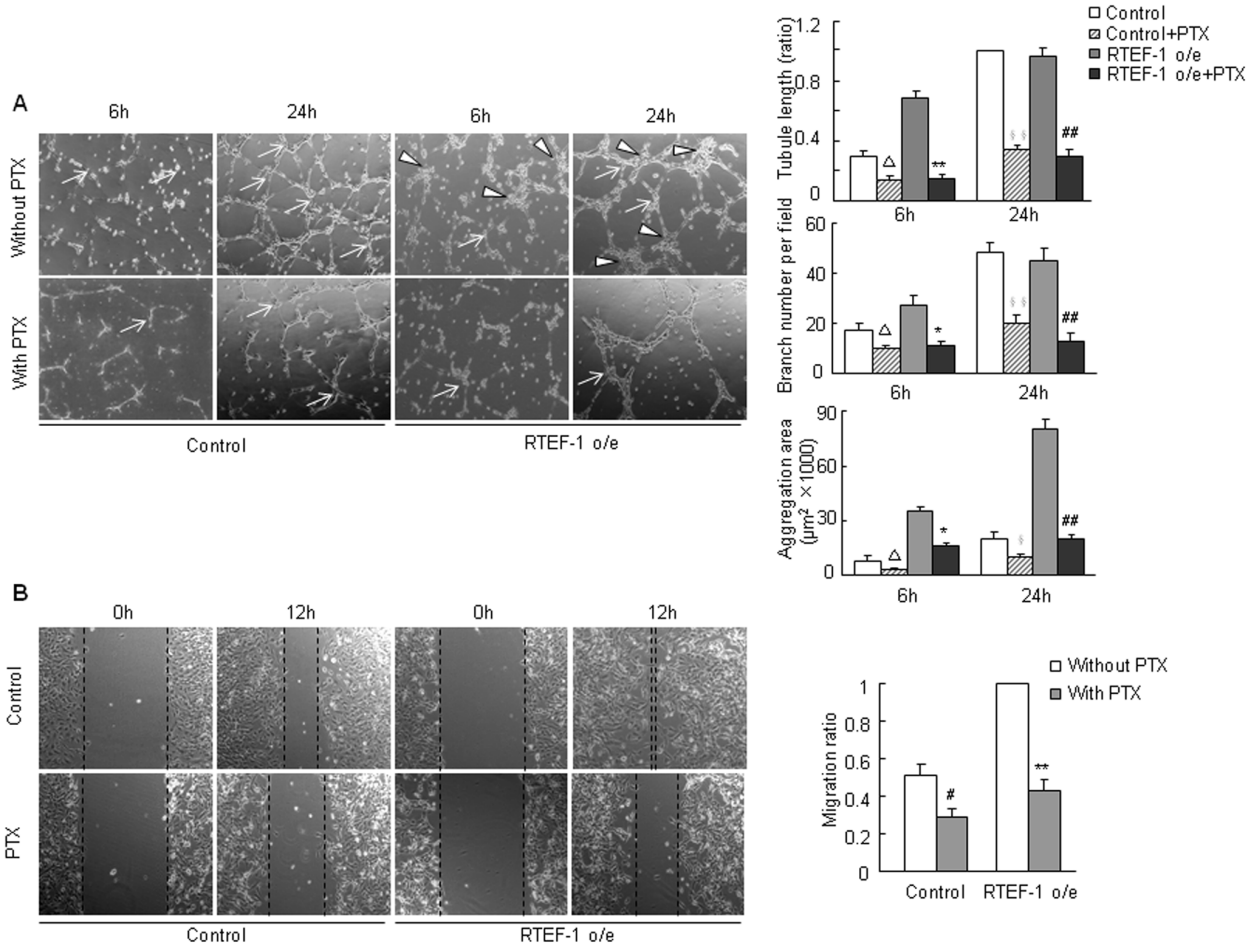
PTX inhibits RTEF-1 driven endothelial cell aggregation and migration. **A.** PTX decreased RTEF-1 driven endothelial cell tube formation and aggregation. (Δ = *p*<0.05, *vs* control HMEC-1 at 6 h Matrigel; * = *p*<0.05, *vs* RTEF-1 o/e HMEC-1 at 6 h Matrigel; ** = *p*<0.01, *vs* RTEF-1 o/e HMEC-1 at 6 h Matrigel; § = *p*<0.05, *vs* control HMEC-1 at 24 h Matrigel; §§* = p*<0.01, *vs* control HMEC-1 at 24 h Matrigel; ##* = p*<0.01, *vs* RTEF-1 o/e HMEC-1 at 24 h Matrigel; Triangle: aggregation area; Arrow: branch) **B.** PTX retarded RTEF-1 driven endothelial cell migration (# = *p*<0.05, *vs* control HMEC-1 without PTX treatment; ** = *p*<0.01, *vs* RTEF-1 o/e HMEC-1 without PTX treatment). The results were quantified based on three experiments and are presented as mean±S.D.

To further confirm that the Edg-1 post-receptor signaling pathway plays a role in RTEF-1 stimulated endothelial angiogenesis, RTEF-1 o/e HMEC-1 and control HMEC-1 cells were treated with or without PTX, a Gi/Go protein inhibitor. The result showed that PTX had an obvious inhibitory effect on tube formation and aggregation in both RTEF-1 o/e HMEC-1 and control HMEC-1 at 6 h and 24 h Matrigel (Figure 4A). Furthermore, treatment with PTX showed a significant retardation of migration in RTEF-1 o/e HMEC-1 and control HMEC-1 (Figure 4B).

## Discussion

Angiogenesis is the physiological process through which new blood vessels form from pre-existing vessels. Sprout angiogenesis generates from the activation of endothelial cells by angiogenic growth factors. The activated endothelial cells proliferate into the surrounding matrix and form solid sprouts connecting neighboring vessels. After that, endothelial cells migrate in tandem and form loops to become a full-fledged vessel lumen. An increase in capillary network formation, endothelial cell proliferation and migration are the most important features of angiogenesis. We have observed an increase capillary formation at quadriceps [3] and heart [14] in VE-Cad/RTEF-1 mice compared with wild type mice in our previous work. It demonstrates that RTEF-1 has a proangiogenesis effect *in vivo*. In this study we found that stable over-expression of RTEF-1 cDNA in endothelial cells enhanced cell growth, migration and capillary network formation, while knockdown of RTEF-1 by its siRNA slowed down these processes of angiogenesis. These data indicate that RTEF-1 plays an important role in angiogenesis by activating endothelial cells *in vitro*.

Downstream genes such as VEGF and Cx43 have been suggested to be involved in RTEF-1 induced angiogenesis [7], [9] and Cx43 has been revealed to involve in RTEF-1-enhanced endothelial cell connections. Quantitative real-time PCR detection showed that RTEF-1 over-experssion increased Cx43 mRNA in HMEC-1 cells at Matrigel. The enhancing effect of RTEF-1 on Cx43 gene expression in Matrigel was observed distinct at 6 h and fell off at 24 h (Figure S2A). However, RTEF-1 over-expression showed an apparent increasing in stimulating endothelial cell tube formation and aggregation in Matrigel at 24 h compared to 6 h (Figure 1C). It indicated that there might be some new genes play a role in this context. Edg-1 has been reported to enhance endothelial cell and vascular smooth muscle cell proliferation and migration and plays a key role in developmental and pathological angiogenesis [17]. Interestingly, we found that Edg-1 mRNA was in a higher level in RTEF-1 o/e HMEC-1 at 24 h Matrigel than at 6 h Matrigel (Figure S2B). Moreover, our data revealed with convincing evidence, that Edg-1 is a target gene of RTEF-1. First, stable over-expression of RTEF-1 cDNA in endothelial cells and transgenic over-expression of RTEF-1 in mice endothelium led to an increase in Edg-1 mRNA and protein levels, while knockdown of RTEF-1 expression by siRNA and endothelial-specific knockout of RTEF-1 in mice caused a decrease in Edg-1 gene expression levels. Second, Edg-1 was detected to be co-localized with RTEF-1 in endothelial cells in vivo. Third, RTEF-1 demonstrated a significant dose-dependent stimulating effect on the Edg-1 promoter upon binding to it. The evidence indicates that Edg-1 is a downstream target gene of RTEF-1.

Whether Edg-1 takes part in RTEF-1-enhanced angiogenesis in endothelial cells is largely unknown. In the current study, we evaluated the effect of Edg-1 on RTEF-1-related endothelial capillary network formation, cell growth and migration. Our data showed that knockdown of Edg-1 expression by its siRNA attenuated the stimulating effect of RTEF-1 on endothelial cell growth, migration and aggregation. Although Edg-1 siRNA did not produce a decreasing tube formation in RTEF-1 o/e HMEC-1, it inhibited tube formation in control HMEC-1. It revealed that knockdown of Edg-1 by siRNA could retard the effect of endogenous RTEF-1 but cannot overcome the over-expression of RTEF-1. As a receptor, the activation or inactivation of Edg-1 might play a more important role in receptor function than expression changes of the receptor itself. Therefore, whether Edg-1 receptor coupled pathways are involved in RTEF-1 driven angiogenesis is needed to be clarified.

Previous investigation revealed that Edg-1 coupled with members of the Gi family (sensitive to PTX) but not Gs, Gq, or G12/13 (insensitive to PTX) [18]. Observations also found that PTX suppressed endothelial cell DNA synthesis and migration in response to S1P, a specific ligand of Edg-1. It suggested that the activated Edg-1 function is mediated through PTX-sensitive Gi/Go –proteins [11]. Our results showed that PTX interrupted RTEF-1 o/e HMEC-1 capillary network formation and migration, indicating that PTX-sensitive Gi/Go -proteins coupled with the Edg-1 pathway are involved in RTEF-1 driven angiogenesis in endothelial cells.

In summary, we have shown for the first time that RTEF-1 binds to the Edg-1 promoter in mature endothelial cells, induces Edg-1 transcription, and is required for endothelial angiogenesis. Edg-1 is a new target gene of RTEF-1 involved in angiogenesis. Furthermore, PTX-sensitive Gi/Go -protein pathway coupled with Edg-1 was discovered to play an important role in RTEF-1 driven angiogenesis in endothelial cells. These findings would be helpful to elucidate the role of RTEF-1 as a transcription factor in angiogenesis and have important potential in development of agents capable of intervening endothelial angiogenesis through RTEF-1 during relevant pathophysiological events.

## Supporting Information

Figure S1 **DNA microarray of RTEF-1 gene knockdown by RTEF-1 siRNA in HUVEC.** Compared to negative control siRNA, knockdown of endogenous RTEF-1 by three different duplexes of RTEF-1 siRNA in HUVEC decreased Edg-1 gene expression. Data was analyzed by Ingenuity Pathway Analysis software (Ingenuity System, Redwood City, CA).
(TIFF)

Figure S2 **Cx43 (A) and Edg-1 (B) mRNA in RTEF-1 o/e and control HMEC-1s in 6** **h and 24** **h Matrigel.** Total RNA was extracted from cell lysates obtained from dissolved cell networks in 6 h and 24 h Matrigel. Quantitative real-time PCR amplification was performed. The results were quantified based on three experiments and are presented as mean±S.D. (* = *p*<0.05, *vs* control 6 h HMEC-1; ** = *p*<0.01, *vs* control 6 h HMEC-1; #* = p*<0.05, *vs* control 24 h HMEC-1; ##* = p*<0.01, *vs* control 24 h HMEC-1).
(TIF)
