# Supplementary figures and images for: Endothelial Differentiation Gene-1, a New Downstream Gene Is Involved in RTEF-1 Induced Angiogenesis in Endothelial Cells
Source: PLoS One. 2014 Feb 10;9(2):e88143. doi: 10.1371/journal.pone.0088143 (PMC3919740; doi:10.1371/journal.pone.0088143)

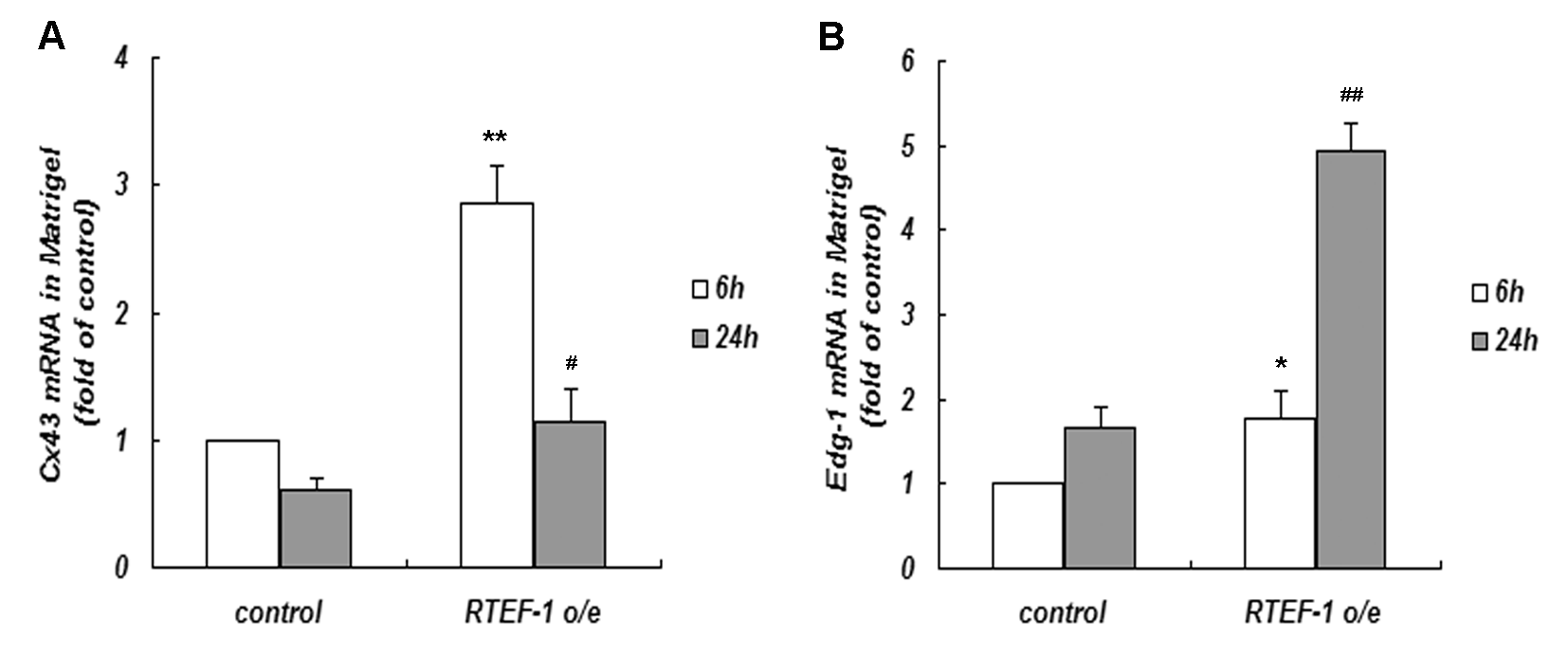

Supplement: Figure S2 — Cx43 (A) and Edg-1 (B) mRNA in RTEF-1 o/e and control HMEC-1s in 6 h and 24 h Matrigel. Total RNA was extracted from cell lysates obtained from dissolved cell networks in 6 h and 24 h Matrigel. Quantitative real-time PCR amplification was performed. The results were quantified based on three experiments and are presented as mean±S.D. (* = p<0.05, vs control 6 h HMEC-1; ** = p<0.01, vs control 6 h HMEC-1; # = p<0.05, vs control 24 h HMEC-1; ## = p<0.01, vs control 24 h HMEC-1). (TIF) [file pone.0088143.s002.tif]
